# Aqueous Acetamiprid Degradation Using Combined Ultrasonication and Photocatalysis Under Visible Light

**DOI:** 10.1007/s11270-022-05867-4

**Published:** 2022-09-24

**Authors:** Carolina Sayury Miyashiro, Safia Hamoudi

**Affiliations:** Department of Soil Sciences & Agri-Food Engineering, Centre in Green Chemistry & Catalysis, Centr’Eau, Université Laval, Québec, G1V 0A6 Canada

**Keywords:** Acetamiprid, Degradation, Ultrasonication, Photocatalysis, Sonophotocatalysis

## Abstract

Acetamiprid (ACE), a neonicotinoid pesticide widely used in pest control, was found in high concentrations in soils, rivers, and lakes. In the present study, ACE degradation was investigated using visible light driven photocatalysis over nitrogen-graphene oxide (N-GO) and palladium-graphene oxide (Pd-GO)–doped ZnO photocatalysts combined with ultrasonication implemented either as a pretreatment (sonolysis) or operated simultaneously with photocatalysis (sonophocatalysis). The effectiveness of the two ACE degradation processes was determined separately. The sonolysis pretreatment allowed reaching almost 40% acetamiprid conversion within 30 min of reaction. Pursuing with the photodegradation reaction in the presence of N-GO-ZnO and Pd-GO-ZnO resulted in a maximum conversion of 98% of ACE within 5 h. As for the sonophotocatalysis process, the reaction time was shortened from 5 to 2 h with 100% acetamiprid conversion. In addition, the photocatalysts were shown to keep their activity even after 5 sonophotocatalytic cycles, thus proving their reusability.

**Graphical abstract:**

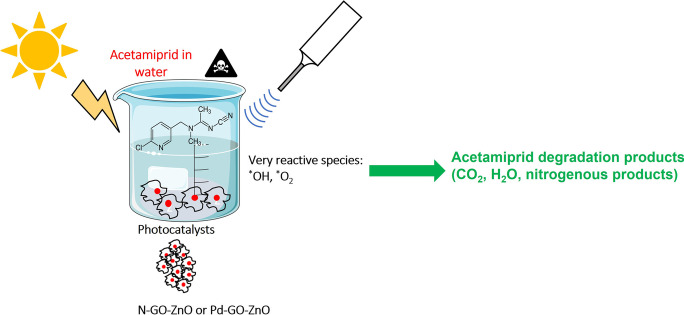

**Supplementary Information:**

The online version contains supplementary material available at 10.1007/s11270-022-05867-4.

## Introduction

Acetamiprid is a systemic insecticide belonging to the neonicotinoid group. It shows excellent efficacy in the control of several insects affecting a wide range of cultures (Hladik et al., 2018). This pesticide is used extensively in foliar sprays. However, only approximately 2–20% of the applied acetamiprid is absorbed by crops, while the remaining enters the environment. Therefore, acetamiprid residues may cause food-chain contamination, threaten the ecosystem, and affect invertebrates (Chakroun et al., 2016). Indeed, this bee-killer pesticide is suspected to induce harmful effects to human and aquatic life (Francisco et al., 2016). Acetamiprid has been often detected in natural bodies of ground- and surface- water and even in drinking water due to its excessive use to enhance crop production (Berny’s et al., 2019; Cruz-Alcalde et al., 2017). Consequently, agricultural applications of acetamiprid are associated with a significant risk to the environment and human health. Hence, the removal of this deleterious pollutant is essential to eliminate or at least minimize its negative impacts (Guo et al., 2019a).

Several physicochemical methods such as high-temperature wet air oxidation (Mishra et al., 1995) and chemical decomposition (Song et al., 2017) were used successfully to eliminate numerous organic compounds from water and wastewaters. Among the various processes carried out for the degradation of organic pollutants, the ultrasonic cavitation and heterogeneous photocatalysis processes are very attractive. The combination of the cavitation method with other advanced oxidation processes offers several advantages over other conventional treatment methods in terms of enhanced degradation efficiencies, reduction in sludge production, less chemicals utilized, and overall reduction in the environmental impact to a greater extent (Babu et al., 2016). The degradation due to cavitation occurs from jets of microbubbles at high speed that collide with the polluting compounds. Also, the cavitation method is a simple process that can be performed under ambient conditions without the help of external chemicals or catalysts (Raut-Jadhav et al., 2013). However, the degradation of wastewater using only ultrasonic cavitation, in the case of a mixture of pollutants, is difficult. To achieve better removal efficiencies, ultrasounds are mainly used in combination with other advanced oxidation processes (Thanekar et al., 2021). Furthermore, heterogeneous photocatalysis mainly relies on the generation of hydroxyl radicals which can convert a wide range of toxic organic compounds into relatively harmless end-products such as CO_2_ and H_2_O. The combined action of semiconductor photocatalyst, an energetic radiation source, and an oxidizing agent leads to the destruction of organic compounds (Fenoll et al., 2015; Pérez et al., 2018).

Zinc oxide (ZnO) is considered among the most common semiconductor photocatalysts due to its great stability, low-cost, non-toxicity, and its convenient synthesis (Ong et al., 2018). However, the implementation of ZnO has been hampered due to the high-speed of combination of the photogenerated electron–hole pairs which inhibits the photocatalytic efficiency. Also, ZnO is usually used as a photocatalyst under ultraviolet (UV) light and exhibits low photocatalytic activity under visible light. Therefore, its widespread application in advanced oxidation processes applied for environmental remediation is less convenient. To circumvent this problem, ZnO doping with noble metals, nitrogen and graphene oxide represents a judicious strategy to enlarge its light absorption to the visible region and concomitantly lower the speed of recombination of the photogenerated electron–hole pairs (Huo et al., 2016; Pathak et al., 2019; Sun et al., 2020a, b).

The present investigation proposes a hybrid approach to remove acetamiprid, a notorious neonicotinoid pesticide, from polluted water through the application of two treatment methods, namely ultrasonication and photocatalysis. Therefore, degradation of acetamiprid in synthetic aqueous solutions was studied using: (i) the ultrasonic cavitation as a pretreatment to heterogeneous photocatalytic process in the presence of ZnO-based photocatalysts under visible light and (ii) simultaneous combination of ultrasonic cavitation and visible light driven photocatalysis (sonophotocatalysis).

## Materials and Methods

### Materials

All reagents were of the highest purity and were used without further purification. Acetamiprid (N-(6-Chloro-3-pyridylmethyl)-N′-cyano-acetamidine) was purchased from Sigma-Aldrich. For the reaction samples extraction and preparation before analysis, acetonitrile (Fisher) as well as magnesium sulfate (MgSO_4_) and anhydrous sodium chloride (NaCl) from Sigma-Aldrich were used.

### Synthesis of the Photocatalysts

The synthesis of ZnO-based photocatalysts was conducted using the precipitation method reported in the work of Perillo et al. (2017) with some modifications. The synthesis of nitrogen-doped zinc oxide (N-ZnO) followed the same procedure of precipitation of ZnO, except that urea as a source of nitrogen was added in the zinc nitrate solution.

The synthesis of palladium-doped zinc oxide (Pd-ZnO) photocatalyst was carried out following the precipitation method reported in the work of Guy et al. (2016) with some modifications. The hydrothermal aging step was performed in a temperature-controlled microwave oven (CEM Phoenix model) at 160 °C during 5 min.

The synthesis of the composites containing graphene oxide (GO) and either N-ZnO or Pd-ZnO was carried out using the procedure reported by Andrade et al. (2019) and Yousaf et al. (2019) with modifications.

The catalysts were characterized using state of the art characterization techniques as previously reported by Miyashiro and Hamoudi (2021 and 2022).

### Sonolytic Tests

For the tests carried out under sonication, an ultrasound generator from Branson Ultrasonics Corporation (450 W Model, 20 kHz, maximum wave amplitude of 210 μm and maximum nominal power of 450 W) was used. The generator is equipped with a high gain probe (2 cm width, 23 cm length). In a typical experiment, a double wall glass reactor was filled with 250 mL of an acetamiprid aqueous solution with a concentration of 15 mg/L. The ultrasonic probe was positioned at the center of the glass reactor aperture to avoid any contact with the glass wall, then dipped in the solution. A temperature-controlled circulating bath was used to control the reactor temperature through the jacket inside the double wall. This sonolytic treatment lasting 30 min was undertaken before the subsequent photocatalytic tests described below.

Calorimetric method was used to determine the power developed by the ultrasound generator during the tests. To this purpose, the rise in temperature of a fixed quantity of water in an insulated container was measured. The actual power output was calculated using the equation according to Plattes et al. (2017).

1 $$\mathrm{Power}\;\left(\mathrm W\right)=\mathrm{CpxM}\;\left(\frac{\mathrm{dT}}{\mathrm{dt}}\right)$$

where *Cp* is the heat capacity of the solvent (J/g °C), M is the mass of solvent (g), *T* is the temperature in the container, and *t* is the time. The power is calculated in Watt (W) units.

### Photocatalytic and Sonophotocatalytic Tests

The photocatalytic activity was investigated as previously reported by Miyashiro and Hamoudi (2021). The acetamiprid initial concentration was fixed to 15 mg/L, the photocatalyst loading to 0.2 g/L, temperature (23 °C), and time interval (0–300 min). The reaction volume was 250 mL for all tests performed under agitation (600 rpm). At preset reaction times, aliquots of the reaction medium were withdrawn, filtered, and analyzed.

**Fig. 1:**
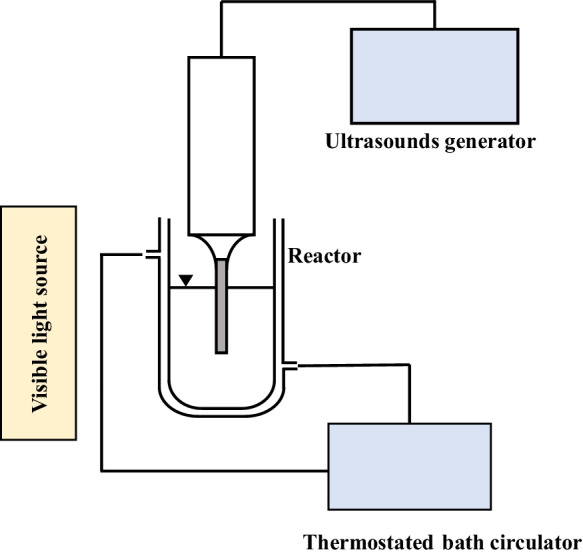
Schematic representation of the sonophotocatalysis experimental setup

The experimental setup used for the tests combining photocatalysis and ultrasounds is presented in Fig. 1. The tests were carried out under the same conditions as described for the photocatalytic tests except that in this case the ultrasonic probe was used.

### Reaction Sample Analysis

All the experiments reported in the present investigation were carried out in duplicate and analyzed twice. The reaction samples were solvent extracted before their analysis by gas chromatography. The extraction was carried out according to the method reported by Suganthi et al. (2018) with slight modification. The gas chromatograph was a Hewlett Packard (HP) 6890 Series gas chromatograph equipped with a flame ionization detector (Agilent ChemStation). The column used was an HP-5 measuring 30 m × 0.25 mm ID × 0.25 mm film thickness. The injector and detector temperature were adjusted to 250 °C, and 1 μL of the sample was injected with a 50:1 split ratio. The oven temperature profile was as follows: initial temperature 100 °C, ramp 10 °C/min at 250 °C, hold for 5 min, ramp 30 °C/min to 300 °C, hold for 5 min. The conversion of acetamiprid during the experiment was calculated according to the following equation.

2 $$\mathrm{Conversion}=\frac{{\mathrm C}_0-{\mathrm C}_{\mathrm t}}{{\mathrm C}_0}\mathrm x100$$

where, *C*_*0*_ and *C*_*t*_ correspond to the initial and the time *t* concentration of the acetamiprid solution, respectively.

## Results and Discussion

### Effect of Ultrasonic Power

The power developed by the ultrasound generator used in the present investigation was measured for the three amplitudes 20%, 50%, and 100% (see Table 1). As seen, a significant increase in the ultrasonic power was reached as the amplitude increased. Also, an increase in temperature was correlated with the amplitude increase. Therefore, as the ultrasounds amplitude was increased, the energy output for the acetamiprid solution increased leading to a higher temperature which in turn helped in the formation of very reactive hydroxyl radicals responsible of the degradation of acetamiprid. Among the tested amplitudes, the greatest degradation of the acetamiprid was obtained with the highest amplitude. Indeed, 39% acetamiprid conversion was reached after 30 min with the highest amplitude (100%). In addition to the increase in hydroxyl radical generation, the increase in amplitude causes an increase in pressure, temperature, and microbubbles formed at high power, which leads to a higher extent of acetamiprid degradation. As reported by Patil et al. (2021), the use of ultrasounds induces high degradation due to the acoustic cavitation phenomenon causing the formation of microjets, turbulence, water cleavage, and generation of more OH^.^ radicals responsible of the organic molecules degradation.

**Table 1:** Effect of amplitude on temperature, energy dissipation, and ACE degradation

Amplitude [%]	Temperature [°C]	Energy dissipation [kW/m^3^]	Degradation ACE [%]^a^
20	21	139	12
50	24	975	25
100	29	2355	39

^a^ACE initial concentration: 15 mg/L; reaction time: 30 min

The observed physical properties of the different photocatalysts investigated in the present work are summarized in Table 2.

**Table 2:** Photocatalysts textural and optical properties

Photocatalyst	Surface area [m^2^/g]	Average pore size [nm]	Pore volume [cm^3^/g]	Band gap energy [eV]
ZnO	30.90	6.38	0.27	3.25
N-GO-ZnO	29.69	1.92	0.13	3.17
Pd-GO-ZnO	49.52	9.53	0.18	3.02

**Fig. 2:**
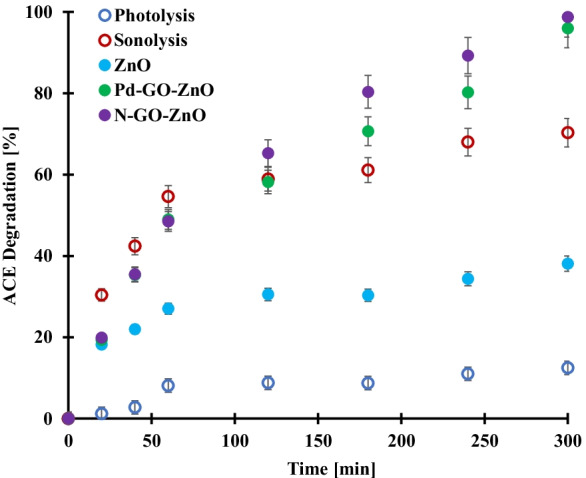
Acetamiprid degradation time profiles under photolysis, sonolysis, and heterogeneous photocatalysis in the presence of ZnO, N-GO-ZnO, and Pd-GO-ZnO (photocatalyst loading: 0.2 g/L); acetamiprid initial concentration: 15 mg/L at room temperature. Error bars denote standard deviation. The tests were duplicated, and the samples were analyzed twice

The comparative degradation time profiles of acetamiprid depicted in Fig. 2 was investigated through the processes of sonolysis, photolysis, and heterogeneous photocatalysis over bare ZnO and doped N-GO-ZnO and Pd-GO-ZnO materials. As seen, in the absence of any catalyst, while photolysis allowed reaching ca. 12% acetamiprid conversion within 5 h of reaction, sonolysis attained 70% acetamiprid conversion within the same time interval. This clearly demonstrates that the pressure and velocity of the cavitation bubbles are sufficient to break down the acetamiprid molecules. The formation, growth, and collapse of gas filled cavities in a liquid are formed in regions where these cavities grow and oscillate in a varying pressure field and subsequently collapse in a region of higher pressure. Collapse of cavities results in localized regions with intense shear, leading to the formation of highly oxidizing hydroxyl radicals (Bai et al., 2017; Yasui et al., 2019). Interestingly, sonolysis outperformed photocatalysis over the bare ZnO photocatalyst. However, the best performances were achieved using photocatalysis over the composites N-GO-ZnO and Pd-GO-ZnO which allowed to completely degrade the acetamiprid within 5 h of reaction under visible light. On the light of these promising results combination of sonolysis with photocatalysis appeared to be a judicious approach to implement in view to get rid of some neonicotinoid pesticides in water.

### Sonolysis as a Pretreatment Before Heterogeneous Photocatalysis

**Fig. 3:**
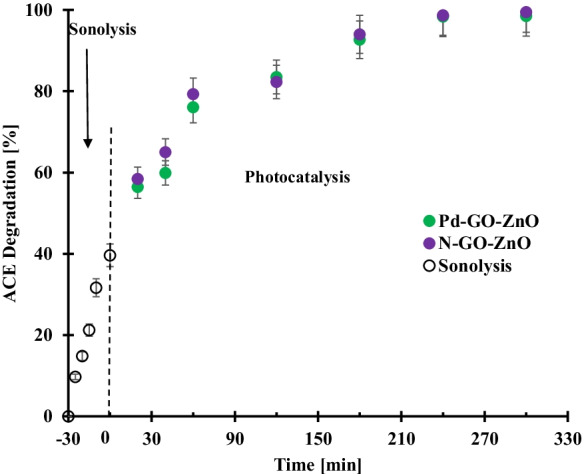
Effect of sonolysis as pretreatment before photocatalysis over N-GO-ZnO and Pd-GO-ZnO for the degradation of acetamiprid. Photocatalyst loading: 0.2 g/L; acetamiprid initial concentration: 15 mg/L at room temperature. Error bars denote standard deviation. The tests were duplicated, and the samples were analyzed twice

Figure 3 depicts the results obtained for the application of ultrasonic cavitation as a pretreatment during 30 min before the photocatalytic degradation of acetamiprid.

As shown, with the ultrasonic cavitation process helping in the degradation of acetamiprid with 40% conversion reached after 30 min, continuing the reaction under visible light irradiation in the presence of N-GO-ZnO and Pd-GO-ZnO photocatalysts allowed achieving almost complete acetamiprid degradation within 4 h. The combination of ultrasonic cavitation with other advanced oxidation processes ensures efficient mineralization of pollutants with better synergistic effects compared to stand-alone advanced oxidation processes (Gore et al., 2014). Besides, only few investigations were reported in the open literature on the combination of sonolysis and photocatalysis under visible light (Madhavan et al., 2019; Panda & Manickam, 2017).

### Sonophotocatalytic Degradation

**Fig. 4:**
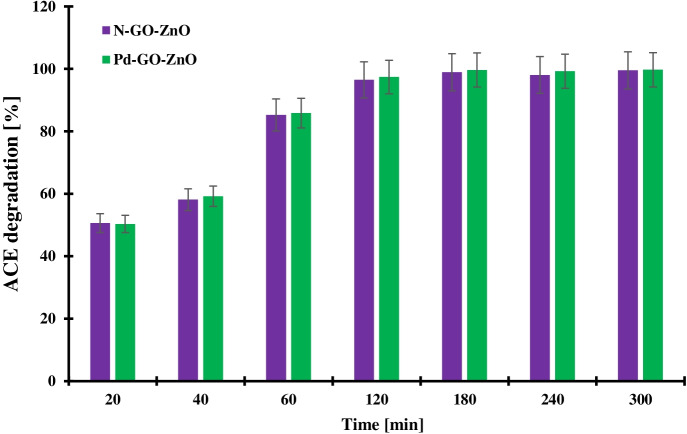
Sonophotocatalytic acetamiprid degradation time profiles in the presence of N-GO-ZnO and Pd-GO-ZnO. Photocatalyst loading: 0.2 g/L; Acetamiprid initial concentration: 15 mg/L at room temperature. Error bars denote standard deviation. The tests were duplicated, and the samples were analyzed twice

The concomitant combination of sonolysis and photocatalysis (sonophotocatalysis) was conducted to optimize acetamiprid degradation in terms of percentage of degradation and reaction duration before reaching complete acetamiprid degradation. The results of this process are illustrated in Fig. 4.

Clearly, the combination of ultrasonic cavitation and photocatalysis over both photocatalysts allowed reaching almost 98% acetamiprid conversion within 2 h of reaction. Under otherwise the same operating conditions 80% acetamiprid conversion was obtained without the application of ultrasounds. Such outstanding performances confirm the effectiveness of combining the two advanced oxidation processes in the degradation of aqueous acetamiprid. Indeed, the application of ultrasonic cavitation effectively benefits the breakdown of acetamiprid molecules in contact with the cavitation bubbles causing the formation of very reactive radicals from the dissociation of water molecules (Gogate & Bhosale, 2013). In this regard, the detailed reactions leading to the generation of these reactive species are summarized in Eqs. (3)–(8) as reported by Theerthagiri et al. (2021):

3 $${\mathrm{H}}_{2}\mathrm{O}+\mathrm{ultrasounds }\to^*H+^{*}{OH}$$

4 $${\mathrm{H}}_{2}\mathrm{O}+^{*}{H }\to {\mathrm{H}}_{2}+^{*}{OH}$$

5 $$^{*}{OH}+^{*}{OH}\to {\mathrm{H}}_{2}{\mathrm{O}}_{2}$$

6 $${\mathrm{O}}_{2}+^{*}{H}\to ^{*}{OH}_{2}$$

7 $${^{*}{OH}}_{2}+{^{*}{OH}}_{2} \to {\mathrm{O}}_{2}+{\mathrm{H}}_{2}{\mathrm{O}}_{2}$$

8 $${\mathrm{H}}_{2}{\mathrm{O}}_{2}+\mathrm{Utrasounds }\to 2^{*}{OH}$$

Moreover, the detailed reactions related to the photocatalytic degradation of organic molecules over a semiconductor are illustrated below (Eqs. 9–16) as reported previously by Seddigi et al. (2015) and Abdurahman et al. (2021):

9 $$\mathrm{Semiconductor}+\mathrm{h\nu }\to {\mathrm{h}}^{+}+{\mathrm{e}}^{-}$$

10 $${\left({\mathrm{O}}_{2}\right)}_{\mathrm{adsorbed}}+{\mathrm{e}}^{-} \to ^{*}{O}^{-}_{{2}}$$

11 $${\mathrm{H}}_{2}\mathrm{O}\to {\mathrm{OH}}^{-}+{\mathrm{H}}^{+}$$

12 $${^{*}{O}^{-}_{{2}}}+{\mathrm{H}}^{+} \to {^{*}{HOO}}$$

13 $${^{*}{HOO}}+{\mathrm{e}}^{-} \to {\mathrm{HOO}}^{-}$$

14 $${\mathrm{HOO}}^{-}+{\mathrm{H}}^{+}\to {\mathrm{H}}_{2}{\mathrm{O}}_{2}$$

15 $${\mathrm{H}}_{2}{\mathrm{O}}_{2}+{\mathrm{e}}^{-}\to 2{^{*}{OH}}$$

16 $${\mathrm{H}}_{2}{\mathrm{O}}_{2}+{\mathrm{h}}^{+}\to {\mathrm{H}}^{+}+{^{*}{OH}}$$

**Fig. 5:**
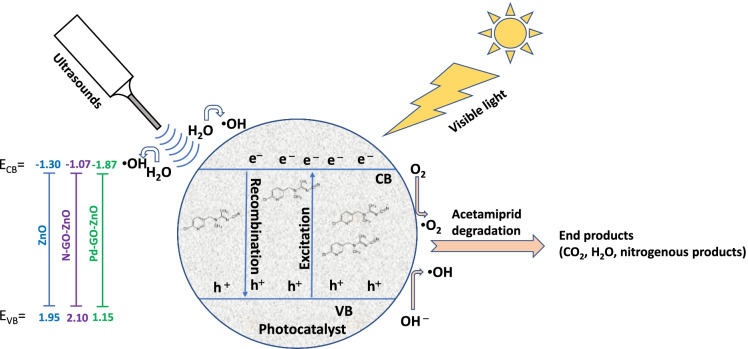
Schematic representation of acetamiprid sonophotocatalytic degradation mechanism

Finally, both reactive species resulting from the sonolysis and photocatalysis react with the pesticide molecule according to the reactions (17) and (18). Accordingly, a schematic mechanism is proposed and illustrated in Fig. 5. As detailed in the supplementary information document, the valence and conduction bands energies illustrated in Fig. 5 were assessed according to the procedures reported by Kamarulzaman et al. (2016); Guo et al. (2019b); Liu et al. (2021); Tayyab et al. (2022).

17 $${^{*}{O}^{-}_{{2}}}+{\mathrm{Acetamiprid}}\to CO_2 + H_2O+{\mathrm{Nitrogenous}\;\mathrm{Products}}$$

18 $${^{*}{OH}}+{\mathrm{Acetamiprid}}\to CO_2 + H_2O+{\mathrm{Nitrogenous}\;\mathrm{Products}}$$

Considering the encouraging findings of the present work, the subsequent scope involves studying the degradation of acetamiprid in actual contaminated surface water stemming from agricultural run-off, groundwater occurring nearby to agricultural fields as well as wastewater effluents from toxin-producing industries. Indeed, the presence of other dissolved chemical species, suspended particles and colloids that may occur in such contaminated aqueous phases can interfere with the degradation process, thus representing an important issue to be addressed.

Furthermore, developing efficient water and wastewater treatment processes is utmost importance to mitigate water pollution and promote water reuse and recycling. Photocatalytic degradation of aqueous acetamiprid over N-GO-ZnO and Pd-GO-ZnO composites proved to be effective (Miyashiro & Hamoudi, 2021, 2022); however, this approach was shown to be time consuming. The present investigation demonstrates eloquently that the hybrid approach integrating photocatalysis and sonolysis is remarkably beneficial as it allowed to drastically shorten the reaction time yielding the complete removal of acetamiprid. Therefore, this hybrid treatment method can be employed successfully in water and wastewater treatment plants to get rid of several organic and emergent pollutants.

### Reuse of N-GO-ZnO and Pd-GO ZnO Catalysts During the Sonophotocatalytic Process

**Fig. 6:**
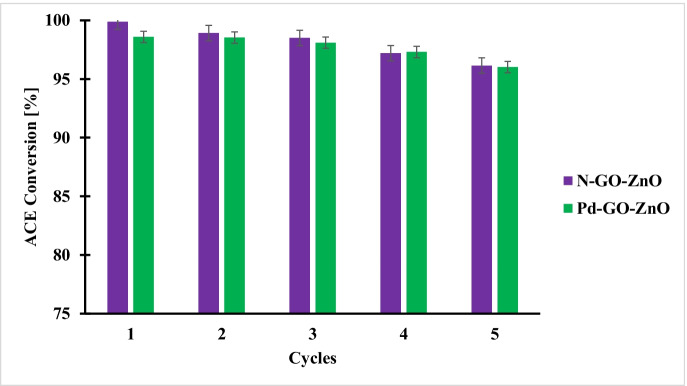
Effect of recycling of the N-GO-ZnO and Pd-GO-ZnO photocatalysts on acetamiprid conversion. Catalyst loading = 0.2 g/L; C_0_ = 15 mg/L; Ultrasounds amplitude 100%; Reaction time = 4 h. Error bars denote standard deviation. The tests were duplicated, and the samples were analyzed twice

Recycling tests were conducted for both photocatalysts to probe their stability for ACE degradation (Fig. 6). The experiments were conducted for 5 continuous cycles. After each run, the catalysts were collected and rinsed several times with distilled water and ethanol and dried at 100 °C for 24 h before the beginning of the next cycle. The results revealed for the two photocatalysts that their performances remained almost unchanged maintaining acetamiprid conversions higher than 96% after 5 successive cycles, thus proving their stability and promising long lasting use without loss of activity.

### Comparison with Other Sonophotocatalytic Findings

Table 3 shows some recent investigations reported in the open literature on the application of the ultrasonic cavitation process combined to photocatalysis for the degradation of organic pollutants in water. In the study reported by Jyothi et al. (2014), application of ultrasounds in combination with irradiation using UV light in the presence of ZnO photocatalyst resulted in 85% phenol degradation. In the report authored by Bokhale et al. (2014), combination of ultrasounds and photocatalysis over TiO_2_ under UV light resulted in a moderate conversion of 63% in the case of Rhodamine 6G after 3 h hours of reaction using a high photocatalyst loading. Besides, Schieppati et al. (2019), Meroni et al. (2020), and Das et al. (2022) investigated the degradation of isoproturon, diclofenac, and methylene blue water pollutants, respectively using sonophotocatalysis over TiO_2_ under UV light. The best performances were obtained for prolonged reaction times (240–360 min). In the reports authored by Kakavandi et al. (2019) and Sun et al., (2020a, 2020b), sonophotocatalytic degradation of tetracycline and Rhodamine B was conducted using TiO_2_ modified using either magnetic activated carbon or bismuth tungstate under UV light. Interesting conversion (90%) was obtained for Rhodamine B while the antibiotic exhibited resistance to degradation after 60 min of reaction. In a very recent investigation, Maridevaru et al. (2022) performed the sonophotocatalytic degradation of the dye Orange 7 over CeNiO_3_ under UV light. Even with a very low pollutant initial concentration (9 mg/L) and a prolonged reaction time, only 74% degradation was reached. As evidenced in Table 3 as well as in the recent review articles (Abdurahman et al., 2021; Deshmukh & Deosarkar, 2022; Panda & Manickam, 2017; Theerthagiri et al., 2021), few sonophotocatalytic investigations were conducted under visible light for the degradation of notorious organic water pollutants, especially pesticides. Interestingly, the present study proved the high efficiency of sonophotocatalysis in the complete degradation of acetamiprid with the application of easy-to-produce photocatalysts and without the addition of any chemical reagents to the process.

**Table 3:** Application of ultrasonication for the degradation of organic pollutants in water

Organic pollutant	Pollutant concent.^1^ [mg/L]	Photocatalyst (loading g/L)	Ultrasounds properties [kHz/W]	Light	Time [min]	Temp [°C]	Degradation %	References
Phenol	40	ZnO (0.1)	40/100	UV	120	29	85	Jyothi et al., 2014
Rhodamine 6G	20	TiO_2_ (4)	50/170	UV	180	25	63	Bokhale et al., 2014
Eriochrome Black T	50	Ti/SBA-15 (1)	20/125	Visible	70	25	89	Gobara et al., 2016
Methylene Blue	20	ZnO/CNT^2^ (0.5)	20/125	Visible	75	25	98	Mohamed et al., 2019
Isoproturon	20	TiO_2_ (0.1)	20/50	UV	240	15	99	Schieppati et al., 2019
Tetracycline	30	TiO_2_/Mag. AC^3^ (0.3)	25/40	UV	60	25	60	Kakavandi et al., 2019
Rhodamine B	20	TiO_2_/Bi_2_WO_6_ (0.5)	35/180	UV	50	25	90	Sun et al., 2020a, 2020b
Diclofenac	25	TiO_2_ (0.1)	20/23	UV	360	45	90	Meroni et al., 2020
Methylene Blue	4.8	TiO_2_ (0.5)	35/480	UV	60	25	80	Das et al., 2022
Orange G	9	CeNiO_3_ (0.2)	38/50	UV	240	25	74	Maridevaru et al., 2022
Acetamiprid	15	N-GO-ZnO (0.2)	20/450	Visible	180	25	100	This work
Acetamiprid	15	Pd-GO-ZnO (0.2)	20/450	Visible	180	25	100	This work

^1^Pollutant initial concentration

^2^CNT: carbon nanotubes

^3^Mag. AC: magnetic activated carbon

## Conclusion

The degradation of aqueous acetamiprid was investigated using ultrasonic cavitation and visible light driven photocatalysis. Employing ultrasonication as a pretreatment before photocatalysis resulted in approximately 40% acetamiprid conversion within 30 min of reaction. Following such pretreatment, the use of N-GO-ZnO and Pd-GO-ZnO composites as photocatalysts under visible light irradiation permitted to reach almost complete acetamiprid conversion within 3 h of reaction at ambient temperature. When the ultrasonication and photocatalysis were carried out simultaneously, the acetamiprid was entirely degraded within 2 h of sonophotocatalytic reaction. Consequently, this highly effective hybrid and environmentally friendly treatment method can be deployed for the treatment of water and wastewaters containing pesticides and even emergent organic pollutants of great concern nowadays. Besides, the performance of the proposed sonophotocatalytic treatment should be tested on actual contaminated surface- and groundwater as well as wastewater effluents.

## Supplementary Information

Below is the link to the electronic supplementary material.

Supplementary file1 (DOCX 44 KB)

## Data Availability

All the data generated or analyzed within the frame of the present investigation are included in this manuscript.
