# Supplementary material for: Aqueous Acetamiprid Degradation Using Combined Ultrasonication and Photocatalysis Under Visible Light
Source: Water Air Soil Pollut. 2022 Sep 24;233(10):401. doi: 10.1007/s11270-022-05867-4 (PMC9508044; doi:10.1007/s11270-022-05867-4)
Supplement: Supplementary file 1 — Supplementary file1 (DOCX 44 KB) [file 11270_2022_5867_MOESM1_ESM.docx]

**Supporting information**

**Aqueous acetamiprid degradation using combined ultrasonication and photocatalysis under visible light**

Carolina Sayury Miyashiro and Safia Hamoudi^[[1]](#footnote-1)^*

Department of Soil Sciences & Agri-Food Engineering; Centre in Green Chemistry & Catalysis; Centr’Eau, Université Laval, Québec, G1V 0A6, Canada.

**Evaluation of the valence and conduction bands energies of the photocatalysts**

X-ray Photoelectron Spectroscopy (XPS) was used to evaluate the valence bands (VB) of the ZnO, N-GO-ZnO and Pd-GO-ZnO photocatalysts. The XPS spectra were recorded using a PHI 5600-ci spectrometer (Physical Electronics, Chanhassen, MN, USA). The main XPS chamber was maintained at a pressure below 8 × 10^−9^ Torr. A standard aluminum X-ray source (e (1486.6 eV) was used at 300 W to record the spectra without charge neutralization. The detection angle was set at 45º with respect to the normal of the surface and the analyzed area was 0.05 cm^2^.

Figure S1(A) presents the obtained valence band levels of ZnO, N-GO-ZnO and Pd-GO-ZnO materials. The valence bands were evaluated according to the procedure reported by Kamarulzaman et al. (2016) and Guo et al. (2019). Therefore, the observed VB energies (E_VB_) were 2.00, 2.45 and 0.95 eV for the ZnO, N-GO-ZnO and Pd-GO-ZnO materials, respectively (See Figure S1(B)). Moreover, the band gap energies (E_G_) were previously evaluated to be 3.25, 3.17 and 3.02 eV for the ZnO, N-GO-ZnO and Pd-GO-ZnO photocatalysts, respectively (See: Miyashiro & Hamoudi, 2021 and 2022). Since E_G_=E_VB_-E_CB_ (Liu et al., 2021), the conduction band energies (E_CB_) were calculated accordingly and reported in Table S1.

**Table S1.** Energies of valence and conduction bands as well as the bandgap for the photocatalysts

| Photocatalysts | E_VB_ (eV) | E_CB_ (eV) | | Eg (eV) | |
| --- | --- | --- | --- | --- | --- |
| ZnO | 1.95 | -1.30 |  | 3.25 |  |
| N-GO-ZnO | 2.10 | -1.07 |  | 3.17 |  |
| Pd-GO-ZnO | 1.15 | -1.87 |  | 3.02 |  |

**Figure S1.** (A) XPS valence band spectra and (B) Valence band energies for ZnO, N-GO-ZnO and Pd-GO-ZnO photocatalysts.

**References**

Guo, W., Zhao, B., Zhou, Q., He, Y., Wang, Z. & Radacsi, N. (2019b) Fe-doped ZnO/reduced graphene oxide nanocomposite with synergic enhanced gas sensing performance for the effective detection of formaldehyde. *ACS Omega*, 4, 10252−10262.

Kamarulzaman, N., Kasim, M.F. & Chayed N.F. (2016) Elucidation of the highest valence band and lowest conduction band shifts using XPS for ZnO and Zn_0.99_Cu_0.01_O band gap changes. *Results in Physics*, 6, 217–230.

Liu, Y., Zhu, Q., Tayyab, M., Zhou, L., Lei, J. & Zhang, J. (2021) Single-atom Pt loaded zinc vacancies ZnO–ZnS induced type-V electron transport for efficiency photocatalytic H_2_ evolution. *Solar RRL*, 5, 2100536.

Miyashiro, C. S. & Hamoudi, S. (2021) Visible light driven photocatalytic degradation of aqueous acetamiprid over nitrogen and graphene oxide doped ZnO composites. *RSC Advances*, 11, 22508.

Miyashiro, C. S. & Hamoudi, S. (2022) Palladium and graphene oxide doped ZnO for aqueous acetamiprid degradation under visible light. *Catalysts*, 12, 709.

Tayyab, M., Liu, Y., Min, S., Irfan, R. M., Zhu Q., Zhou, L., Lei J. & Zhang, J. (2022) Simultaneous hydrogen production with the selective oxidation of benzyl alcohol to benzaldehyde by a noble‐metal‐free photocatalyst VC/CdS nanowires. *Chinese Journal of Catalysis*, 43, 1165-1175.

1. *Corresponding author: Phone (418) 656 2131 ext: 408460; Fax (418) 656 3723; email: [safia.hamoudi@fsaa.ulaval.ca](mailto:safia.hamoudi@fsaa.ulaval.ca) [↑](#footnote-ref-1)
